# *Streptococcus pyogenes* vaccine candidates do not induce autoimmune responses in a rheumatic heart disease model

**DOI:** 10.1038/s41541-023-00604-2

**Published:** 2023-02-04

**Authors:** Simone Reynolds, Rukshan Ahamed Mohamed Rafeek, Adam Hamlin, Ailin Lepletier, Manisha Pandey, Natkunam Ketheesan, Michael F. Good

**Affiliations:** 1 Institute for Glycomics, Griffith University, Southport, Queensland Australia; 2 School of Science & Technology, University of New England, Armidale, New South Wales Australia

**Keywords:** Peptide vaccines, Preclinical research

## Abstract

We have developed a candidate vaccine to protect against multiple strains of *Streptococcus pyogenes* infections. The candidate vaccine contains two synthetic peptides derived from *S. pyogenes* proteins: the M-protein epitope, p*17 and the IL-8 degrading *S. pyogenes* Cell-Envelope Proteinase (SpyCEP) epitope, K4S2. In this study we utilise a rat autoimmune valvulitis model that displays both the cardiac and neurobehavioural pathology associated with post-streptococcal sequelae, to assess if the vaccine candidate antigens induce autoimmune complications and inflammatory pathology. Each antigen was conjugated to carrier protein diphtheria toxoid (DT) and independently assessed for potential to induce autoimmune pathology in female Lewis rats. Rats were administered three subcutaneous doses, and one intranasal dose over a four-week study with a two-week recovery period. A positive control group received recombinant *S. pyogenes* M5 (rM5) protein, and the negative control group received PBS. Rats that received rM5 developed significant cardiac and neurological pathologies. There was no evidence of these pathologies in the PBS control group, or the rats administered either P*17-DT or K4S2-DT. This study provides further preclinical evidence of the safety of the vaccine candidates p*17 and K4S2 and their appropriateness as candidates in human clinical trials.

## Introduction

*Streptococcus pyogenes* (*S. pyogenes*) is a serious human pathogen effecting people of different ages and socio-economic levels. It causes a diverse range of diseases, including pharyngitis and skin infections (particularly common in children), severe invasive infections, and post-streptococcal sequelae including acute post-streptococcal glomerulonephritis (APSGN), acute rheumatic fever (ARF), rheumatic heart disease (RHD) and Sydenham’s chorea (SC). A review of the global burden of *S. pyogenes* disease published by the World Health Organization^1^ estimated that approximately 18.1 million people suffer from serious *S. pyogenes* disease and that another 1.78 million new cases occur each year. The burden of non-invasive *S. pyogenes*-associated diseases such as pyoderma and pharyngitis were estimated at 111 million cases of pyoderma and 616 million new cases of pharyngitis each year. Existing cases of RHD were estimated at approximately 15.6 million, with 460,000 new RHD cases and 349,000 RHD-related deaths each year^2^. The majority of RHD disease cases occur in developing nations. Indigenous populations in developed countries suffer a disproportionately high rate of streptococcal disease. Indigenous Australians experience one of the highest documented rates of ARF and RHD in the world. From 2014 to 2018 ARF diagnoses increased from 71 to 100 cases per 100,000 and new RHD diagnoses were recorded at a rate of 60 cases per 100,000 among Indigenous Australians^3^. There is currently no vaccine available.

We have developed a candidate vaccine to protect against multiple strains of *S. pyogenes* infections. The candidate vaccine contains two synthetic peptides: p*17 and K4S2. Minimal epitope p*17 is derived from the conserved region of the M-protein, found on the surface of *S. pyogenes* and epitope K4S2 is derived from the IL-8 degrading virulence protein *S. pyogenes* Cell Envelope Proteinase, SpyCEP. Preclinical data have demonstrated that P*17 + K4S2 vaccines formulated with adjuvants Alhydrogel (Alum) or CAF^®^01 are immunogenic and elicit a robust antibody response able to protect immunised animals against lethal challenge with multiple strains of *S. pyogenes*^4,5^.

Our intention is to determine safety, immunogenicity and efficacy of this vaccine in human clinical trials. To do this, evidence of the candidate vaccine’s safety and toxicity must be provided. This is particularly relevant for M-protein-based vaccines. Although M-proteins contain protective (opsonic) epitopes, a challenge to *S. pyogenes* vaccine development has been the identification and avoidance of epitopes that are immunologically cross-reactive with human tissues and/or promote proliferation of cross-reactive T-cells^6–10^. The induction of cross-reactive T-cells is believed to drive the development of post-streptococcal autoimmune sequelae.

We have previously reported on a repeated dose toxicity study in healthy Sprague-Dawley rats, which demonstrated that intramuscular administration of P*17 + K4S2/Alum is well tolerated with no toxicologically adverse findings noted throughout the study^5^. A parallel repeated dose toxicity study of P*17 + K4S2/CAF^®^01 administered intramuscularly + intranasally, also revealed the formulation conferred no adverse toxicological effects^4^. Extending on from the toxicology studies we wished to determine the potential of each vaccine candidate antigen to induce autoimmune or inflammation pathology, to further assess safety of the combination vaccine. To do this we utilised a unique rat autoimmune valvulitis (RAV) model that displays both the cardiac and neurobehavioral pathology associated with post-streptococcal sequelae^11–13^. This model uses female Lewis rats that have a propensity to develop autoimmune valvulitis following exposure to recombinant *S. pyogenes* M-protein^11^.

Here, we report a preclinical study in the RAV model, of the lead candidate vaccine antigens, p*17 and K4S2 conjugated to DT (P*17-DT, K4S2-DT). We tested these candidates for the possibility to initiate an autoimmune response leading to neurobehavioral changes or cardiac abnormalities, using the RAV model. The data demonstrates that these vaccine candidate antigens do not induce autoimmune or inflammatory pathology in the RAV model and provides further evidence of the safety of the vaccine candidate.

## Results

### Observations of neurobehavioural changes associated with post-streptococcal sequelae

We employed the well-characterised RAV model, developed to mimic functional and pathological symptoms of acute rheumatic fever and rheumatic heart disease to assess safety of the vaccine candidate antigens^11^. Previously, Good Laboratory Practice (GLP) toxicological studies with the antigens in combination, conducted in healthy Sprague-Dawley rats, observed no significant toxicological effects^4,5^. Expanding from these assessments, we sought to evaluate the vaccine candidate antigens in isolation and in the context of an ARF/RHD susceptible disease model. Lewis rats (*n* = 6 female) were administered a 200 µL subcutaneous injection of 150 µg P*17-DT, 150 µg K4S2-DT, PBS (negative control) or 500 µg recombinant *S. pyogenes* M5 (rM5) protein (positive control) mixed 1:1 in Complete Freund’s Adjuvant at day 0. At days 1 and 3, rats were injected intraperitoneally with 0.3 µg of *Bordetella pertussis* toxin as an additional adjuvant. On days 7 and 14 all rats received 200 µL booster injections of antigen (150 µg P*17-DT, 150 µg K4S2-DT, PBS (100 µL), 500 µg rM5) mixed 1:1 with incomplete Freund’s adjuvant (IFA), and a final 200 µL booster was administered on day 21 as follows; rM5 (500 µg) and PBS (in IFA) were injected subcutaneously into the flank region and P*17-DT and K4S2-DT (150 µg of each in Tris) were administered intranasally. Rats were euthanized two weeks post final boost (day-35).

**Fig. 1:**
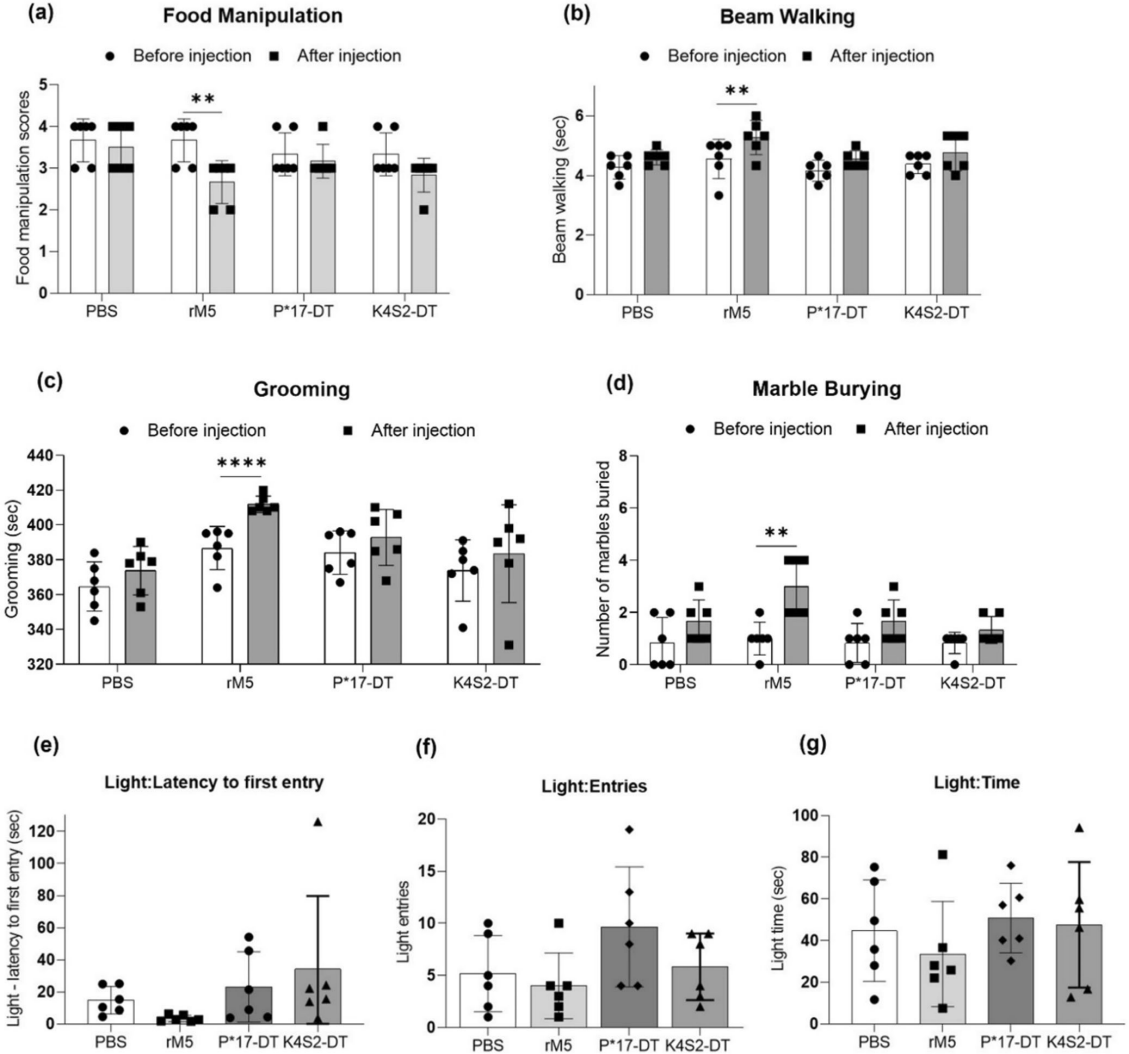
Neurobehavioural assessments. Neurobehavioural changes were assessed in Lewis rats before (•) and after injection of (▪) PBS, rM5, P*17-DT and K4S2-DT using the following tests: **a** food manipulation, **b** beam walking, **c** grooming, **d** marble burying and **e**–**g** light/dark box test (LDB). LDB was performed after injection of PBS (•), rM5 (▪), P*17-DT (▴) and K4S2-DT (♦). In each experiment (*n* = 6), age-matched rats were used. Statistical analysis performed by two-way ANOVA. Significance to control group, *P < 0.05, **P < 0.01, ***P < 0.001, ****P < 0.0001. Error bars represent standard deviation.

Changes to neurobehaviour were assessed in all treatment groups prior to immunisation regimen (before injection) and prior to cull (after injection). Behavioural tests were performed to assess impairment in fine motor control (food manipulation), gait and balance (beam walking), obsessive-compulsive behaviour (grooming and marble burying) and anxiety-like behaviour (light and dark box test). When comparing before and after injection the rM5-treated rats displayed significant changes in food manipulation, grooming and marble burying behaviours. In addition, the time taken to traverse the beam increased and two rats in the group had a single-foot slip (Fig. 1a–d). Conversely, no significant behavioural changes were observed in the P*17-DT, K4S2-DT or PBS treatment groups. To determine if anxiety or changes to exploratory activities were a factor contributing to the observed behaviour, light and dark box tests were performed. Latency to first entry to the light compartment, the number of entries to the light compartment and time spent in the light compartment were recorded (Fig. 1e–g). There were no significant differences observed in any of the treatment groups.

### Assessment of functional and histological abnormalities in cardiac tissue

**Fig. 2:**
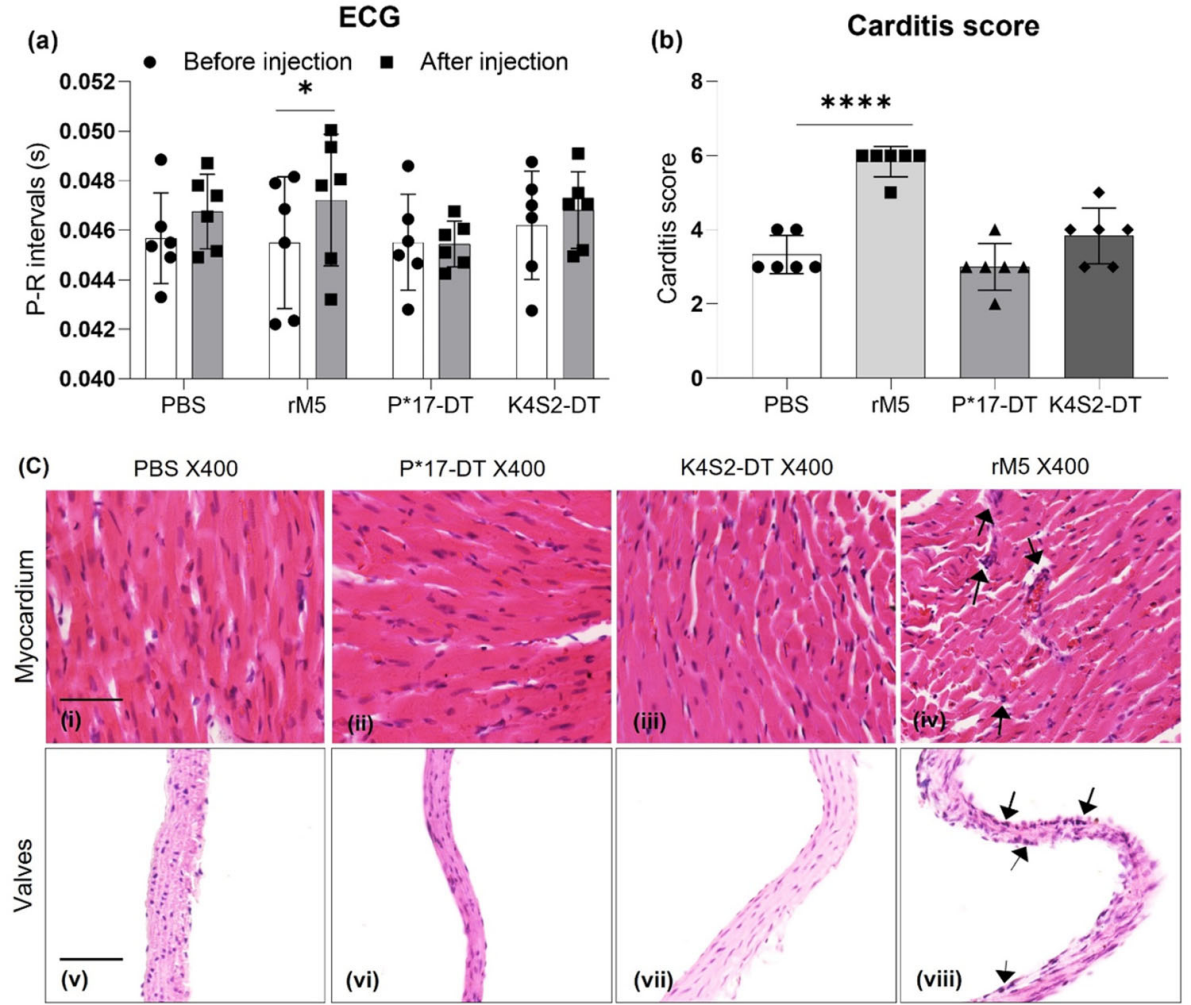
Functional and pathological assessment of the heart. Functional impairment of the heart before (•) and after injection of antigen (▪) was assessed by ECG (**a**). Statistical analysis performed by two-way ANOVA. Significance to before injection shown, *P < 0.05, **P < 0.01, ***P < 0.001. Development of carditis (**b**). Inflammatory changes in the myocardium and valvular tissue, characterised by mononuclear cell infiltration, were scored in PBS (•), rM5 (▪), P*17-DT (▴) and K4S2-DT (♦) treated rats. Statistical analysis performed by one-way ANOVA. Significance to PBS group shown, *P < 0.05, **P < 0.01, ***P < 0.001, ****P < 0.0001. Error bars represent standard deviation. Histological changes in cardiac tissues following exposure to PBS, P*17-DT, K4S2-DT and rM5 (**c**). Arrows indicates the possible mononuclear cell infiltration. Representative images from each group shown, scale bar represents 50 µm.

An important characteristic of the valvulitis model is its ability to induce observable functional and histological changes to the heart, mirroring what is clinically observed in ARF/RHD patients. To determine heart function after treatment, all rats underwent an Electrocardiography (ECG) examination prior to euthanasia (after injection). Baseline heart function prior to treatment (before injection) was used to compare functional changes. A prolongation of the P-R interval was found in the rM5-treated rats indicating a conduction abnormality of the heart. In contrast, P*17-DT and K4S2-DT treated rats demonstrated no change to P-R interval compared to PBS control (Fig. 2a).

Histological staining of the myocardium and mitral valves to detect mononuclear cell infiltrates was used as a means of observing cardiac inflammation (2c). There was minimal evidence of mononuclear cell infiltration in the mitral valves and myocardium in PBS control rats (i, v) and rats treated with P*17-DT (ii, vi) or K4S2-DT (iii, vii). An increased mononuclear cell infiltration was observed in rats injected with rM5 (iv, viii) compared to PBS control group, indicating inflammatory changes in this group. Inflammation of the heart was then ascribed a carditis score based on the number of mononuclear cell infiltrates and focal lesions in both the myocardium and mitral valve (Fig. 2b). Carditis scoring was undertaken using a scoring matrix previously developed^14^ (Supplementary Table 1). Significantly higher carditis scores were observed in rats that received rM5 compared to PBS control and both vaccine treatment groups. Minimal inflammatory cell infiltration was observed in rats that received PBS, P*17-DT or K4S2-DT, however, there was no significant difference in the cell infiltration between these groups.

### Induction of antigen-specific immune responses and cross-reactive M-protein antibodies

**Fig. 3:**
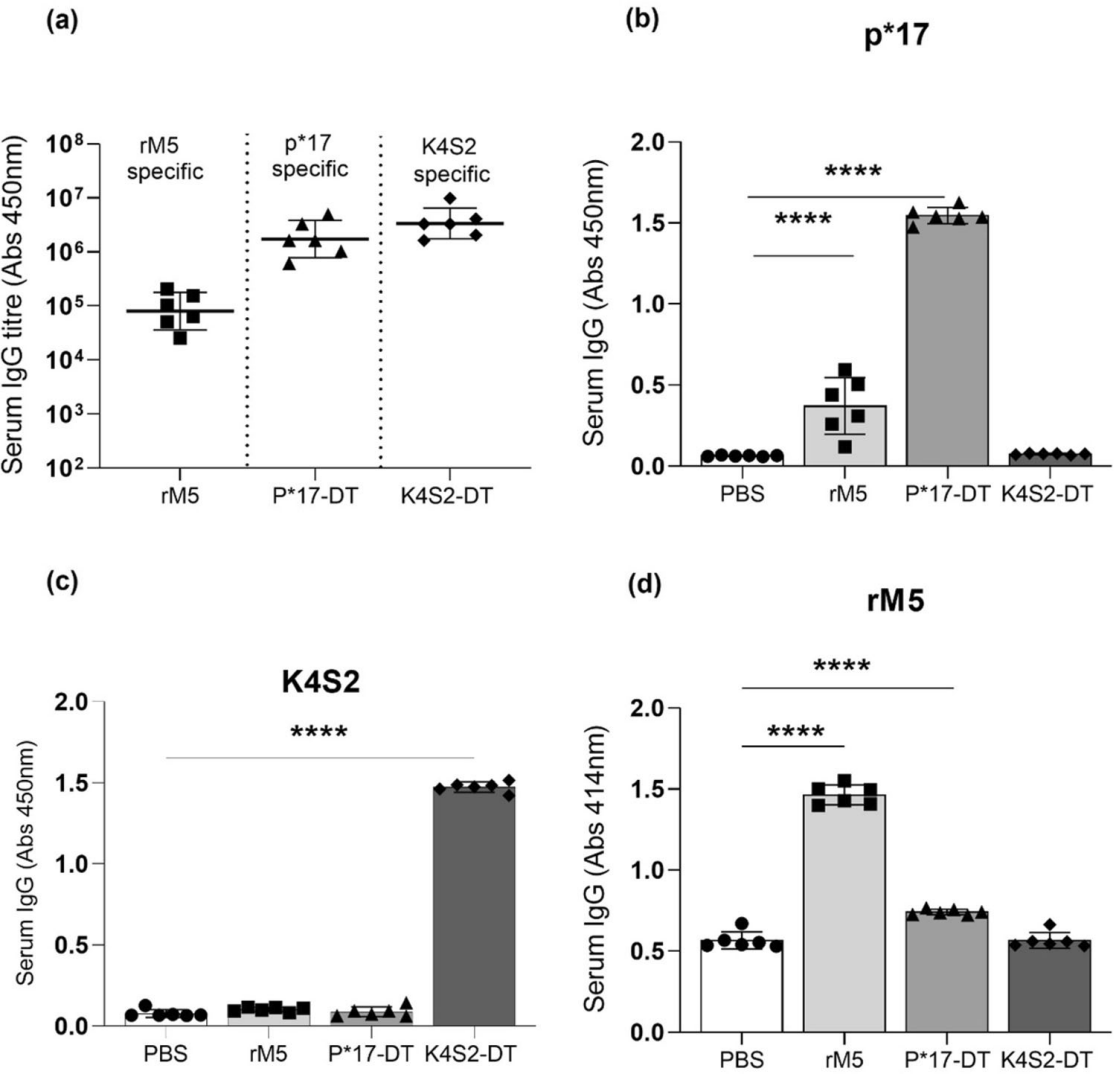
Antibody responses to specific antigen. Antigen-specific serum IgG titres (**a**) and serum IgG reactivity in rats administered with PBS (•), rM5 (▪), P*17-DT (▴) or K4S2-DT (♦) to peptide p*17 (**b**), peptide K4S2 (**c**) and rM5 (**d**). Absorbance values of day-35 rat sera at 1:400 (**b**, **c**) and 1:100 (**d**) is shown. IgG titres were defined as the highest dilution of serum for which OD was >3 standard deviations above the mean OD of PBS control at highest dilution. Statistical analysis performed by one-way ANOVA with Dunnett multiple comparison test. Significance to PBS control group, *P < 0.05, **P < 0.01, ***P < 0.001 and ****P < 0.0001. Error bars represent standard deviation.

An immunological assessment of sera was conducted to confirm the generation of antigen-specific antibodies and assess for cross-reactivity. P*17-DT, K4S2-DT and rM5 treated groups were compared to the PBS negative control group. The in vitro assays confirmed the generation of robust antigen-specific antibodies by all rats within a group (Fig. 3a). In addition, sera from each group were evaluated for antigen cross-reactivity. Significant cross-reactivity of rM5 antisera for p*17 antigen and P*17-DT antisera for rM5 was evident (Fig. 3b, d). K4S2 was specific and showed no cross-reactivity for any other antigen (Fig. 3c).

### Detection of antibodies cross-reactive for cardiac or neuronal tissue proteins

**Fig. 4:**
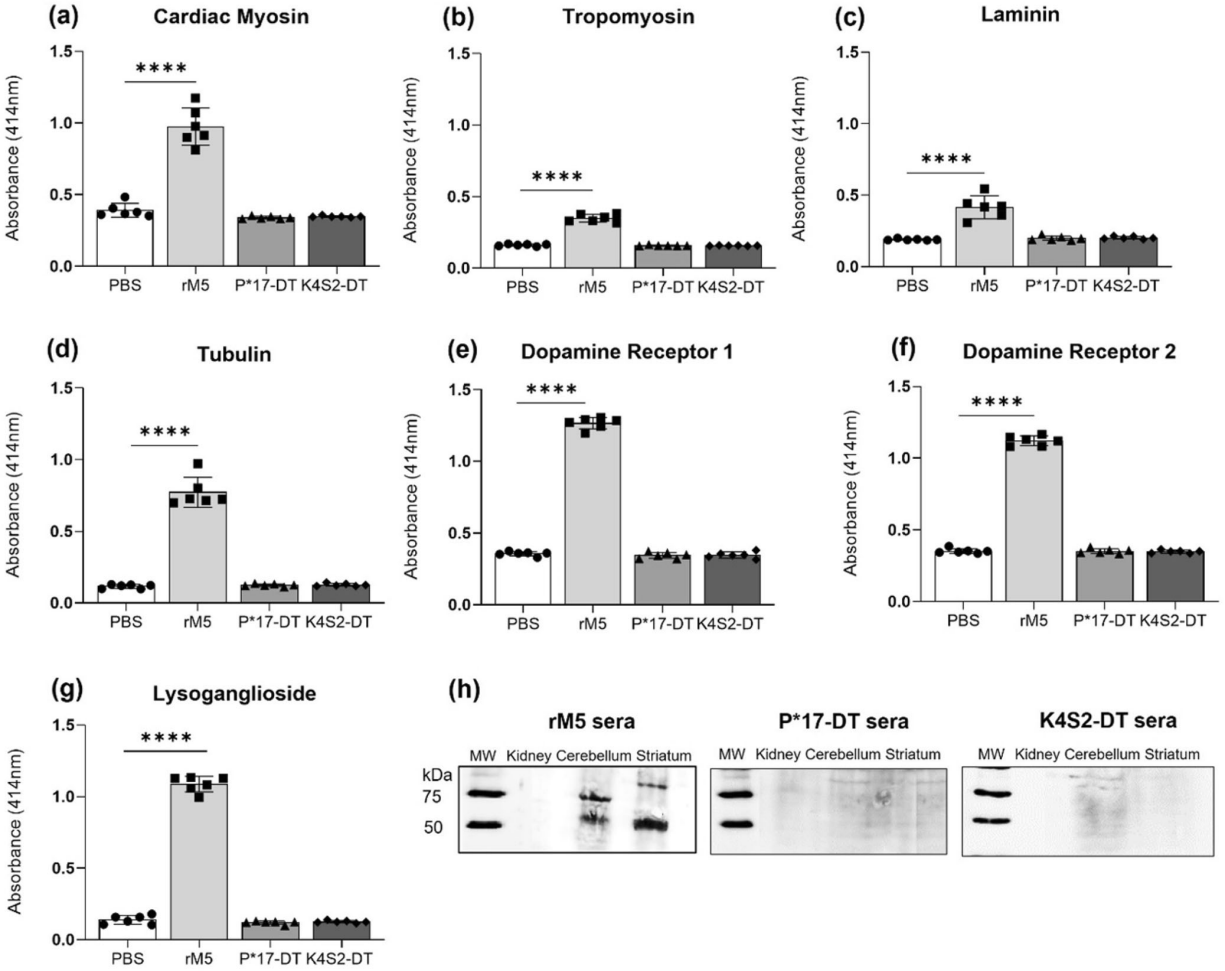
Serum IgG cross-reactivity to host cardiac and neuronal tissue proteins. Serum IgG titre against cardiac myosin (**a**), tropomyosin (**b**), laminin (**c**), tubulin (**d**) dopamine receptor 1 (**e**) and 2 (**f**), and lysoganglioside (**g**) in rats (*n* = 6) injected with PBS (•), rM5 (▪), P*17-DT (▴) or K4S2-DT (♦) are shown. Absorbance values of day-35 rat sera at 1:400. Statistical analysis performed by one-way ANOVA with Dunnett multiple comparison. Significance to PBS group, *P < 0.05, **P < 0.01, ***P < 0.001 and ****P < 0.0001. Error bars represent standard deviation. Serum IgG cross-reactivity to endogenous proteins in lysates of cerebellum and striatum from naive rats (**h**). MW molecular weight.

An important feature of the RAV model is the generation of antibodies that are cross-reactive for host tissue proteins implicated in the development of ARF/RHD^11^. This important attribute models what is observed in patients with RHD. This is believed to induce autoimmunity and subsequent autoimmune pathology^15^. A safety issue for any *S. pyogenes* vaccine is the potential for candidate antigens to induce undesirable autoimmune pathology. It is therefore vital to demonstrate that candidate antigens do not induce antibodies that are cross-reactive with host proteins. To investigate the cross-reactive or autoimmune potential in vitro assays were performed against a panel of proteins present in cardiac and neuronal tissues. Proteins selected represent host tissue proteins for which the development of autoantibodies has been implicated in ARF/RHD^16^. Target proteins for evaluation were myosin and tropomyosin (heart muscle), laminin (extracellular matrix of the heart valves) and tubulin, dopamine receptors and lysoganglioside (brain). Significant cross-reactivity to all tissue proteins was evident in the rM5-treated group compared to the negative control PBS group. Importantly, there was no cross-reactivity observed for the antisera from either the P*17-DT or K4S2-DT groups (Fig. 4a–g). Serum IgG cross-reactivity to host endogenous proteins in brain tissue was also assessed by western blot (Fig. 4h). Antibodies in pooled sera from rats injected with rM5 bound to lysates of cerebellum and striatum from naive rats, pooled sera from rats injected with P*17-DT or K4S2-DT did not bind. Bands on blots observed at ~55 kDa, and ~70 kDa are consistent with the known molecular weights of dopamine receptors 1 and 2, lysoganglioside-_GM1_, and tubulin^17,18^. Sera did not recognise proteins in kidney lysates used as non-neuronal tissue control. GAPDH was used to determine the expression of housekeeping protein in all tissue.

### Detection of cytokine IL-17A in serum and cardiac tissue

**Fig. 5:**
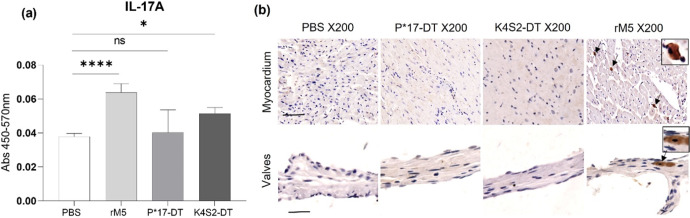
Systemic and localised presence of IL-17A. Detection of pro-inflammatory IL-17A in the sera and cardiac tissues of rats injected with PBS, rM5, P*17-DT or K4S2-DT. IL-17A in sera was assessed by ELISA (**a**). Absorbance values at day-35 are shown. Statistical analysis performed by one-way ANOVA with multiple comparison test. Significance to PBS control group, *P < 0.05 and ****P < 0.0001. Error bars represent standard deviation. Tissue-specific localisation of IL-17A in the myocardium and valvular tissues of rats was investigated by immunohistochemical staining (**b**). Representative images from two rats in each group given (**b**, S1). Images magnified at ×200, snippets magnified at ×400, and scale bar represents 50 µm.

Interleukin-17A (IL-17A) is a major mediator of tissue inflammation in many autoimmune diseases and we have previously shown that this cytokine induces autoimmune carditis in the Lewis rat model of rheumatic heart disease^14^. In the present study, we evaluated if the candidate vaccine antigens could induce undesirable autoimmune pathology associated with elevated IL-17A responses. To investigate this, we first performed an IL-17A ELISA with sera from rats treated with rM5, P*17-DT, K4S2-DT or PBS control group (Fig. 5a). Highly significant IL-17A production was evident in the rM5-treated rats compared to the negative control PBS group. There was no difference observed for the sera from P*17-DT immunised rats in relation to PBS group whilst a small, yet significant increase in the levels of sera IL-17A was observed in K4S2-DT groups. We then explored if IL-17A was evident in cardiac tissues of treated rats (Fig. 5b, Supplementary Figure 1). IL-17A was detected in the myocardium and valvular tissue of rM5-treated rats. No evidence of IL-17A infiltration was observed in the PBS, P*17-DT and K4S2-DT treated rats.

## Discussion

It is imperative that any *S. pyogenes* candidate vaccine be evaluated for its safety. We have previously reported on two repeated dose toxicity studies in healthy Sprague-Dawley rats, which demonstrated that administration of P*17 + K4S2 formulated with Alum or CAF^®^01 are well tolerated with no toxicologically adverse findings noted throughout either study^4,5^. In line with those studies, we wanted to extend the safety considerations to include the potential to induce autoimmune pathology. This relates to clinical observations and a growing body of preclinical evidence that post-streptococcal sequelae like ARF, RHD and the neurobehavioural condition Sydenham’s chorea, have an autoimmune aetiology^19–21^. The current study has enabled us to assess if the vaccine candidate antigens p*17 and K4S2 have a potential role in this aetiology in the context of both cardiac and neurobehavioural changes.

This is the first study to determine if any vaccine candidate causes neurobehavioural changes or cross-reactive antibodies to neuronal tissues suspected to be involved in the autoimmune aetiology of post-streptococcal sequelae. Neurobehavioural assessments of rats administered P*17-DT or K4S2-DT indicated no impact on the behaviour of the rats and the results of the cross-reactive antibody assays further demonstrated no induction of a cross-reactive response to neuronal tissue proteins. The importance of and the demonstrated ability to perform the neurological assessments highlighted in this study, raises the question and potential for the assessment of the same neurological markers in future clinical trials. Just as it has in this study, it would provide an additional tool for assessing potential toxicological impacts of a candidate vaccine and could be a parameter for assessing safety in humans.

The potential of an antigen derived from the M-protein to induce cardiac pathology is a legitimate concern that must be addressed. We have previously investigated the potential of the p*17-related vaccine candidate J8-DT to induce an autoimmune response in a rat model of valvulitis/carditis^22^. The study demonstrated that J8-DT did not induce cardiac lesions. Subsequent to the J8-DT study, another group tested the peptide J14, which contains 12 of the 20 amino acids present in peptide p*17. This study found that J14 also did not induce cardiac lesions^23^. Given that the J8 and J14 epitopes are derived from the same C-repeat region of the M-protein as the p*17 peptide we did not expect P*17-DT to induce cardiac pathology. We also did not expect K4S2-DT to induce cardiac pathology as SpyCEP, has not been implicated in the autoimmune aetiology of RHD or other post-streptococcal sequelae. In addition, previous work has demonstrated that P*17 and K4S2 vaccines did not induce antibodies cross-reactive for cardiac proteins^5,24^. The current study confirmed that immunisation with P*17-DT or K4S2-DT, in a formulation known to induce autoimmune pathology using a recombinant protein as antigen, does not generate antibodies that cross-react with human cardiac proteins or induce inflammation in cardiac tissues.

IL-17A is an inflammatory cytokine that has a dual role in the context of immunity against bacterial infections. At the same time that IL-17 acts upon tissue cells to release IL-8, which in turn recruits neutrophils that then control bacteria, IL-17 and the cells that produce it appear to be involved in priming and exacerbating inflammatory immune response in various tissues including the heart^25,26^. Previous studies using this model have investigated the role of IL-17A in the pathogenesis of ARF and RHD^14,27^. In the context of this model, we wanted to determine if cytokine IL-17A was detectable in the serum and cardiac tissue of vaccinated animals. Although no functional assessment was performed to unravel the mechanistic role of IL-17A, we hypothesise that K4S2 induces the protective element of IL-17A through increase of antibodies that protect host IL-8 from SpyCEP-mediated proteolysis, thus enabling neutrophil bactericidal actions^28^. In contrast, rM5 induces a stronger IL-17A response associated with damage of the heart tissue, as evidenced by IL-17A staining in cardiac tissues, inflammation in the mitral valve and myocardium and a prolonged P-R interval. Thus, it is possible to envisage that a threshold in IL-17 levels can be established to separate protective from pathogenic responses in the context of *S. pyogenes* pathogenesis and vaccine development. Further work will need to be done to extrapolate the immunological pathways at play here.

We note that although the immunisation schedule is the same for all groups, the route of administration for the final dose differs between rM5 and the test groups (P*17-DT, K4S2-DT), subcutaneous injection with IFA versus intranasal administration with Tris. As there is a difference in the final administration route between rM5 and the test groups, this may impact on the differences in findings observed between rM5 and the vaccine antigen groups. The administration of the third booster as an intranasal inoculum, allowed us to see if the vaccine antigens would have an adverse effect that was measurable. We did not expect an adverse outcome to occur, as healthy rats intranasally administered the same antigens in Tris, in a toxicology study, showed no adverse reaction when measured as local irritation, immunogenicity, functionality of antibody, behavioural changes, biochemistry, organ histology or gross pathology, compared to control^4^. The proposed route of administration and schedule intended for a vaccine containing the tested candidate antigens is three intramuscular injections, three weeks apart or two intramuscular and one intranasal administration three weeks apart, also known as the prime-pull strategy. Therefore, it should also be noted that for the vaccine antigen, in the context of its intended clinical usage, the model schedule represents a repeated exposure to antigen that is exaggerated or acute in both time between exposures (one versus three weeks) and number of boosters (three versus two). As such, we would anticipate that an acute administration of the vaccine’s antigens in an autoimmune susceptible animal would induce measurable pathology, if the antigens were auto-antigenic.

To be considered a functionally relevant and useful vaccine in humans, suitable for the prevention of *S. pyogenes* disease, a vaccine should demonstrate three essential features, [1] be multivalent, protecting against several *S. pyogenes* strains, [2] provide long-lasting immunity, including appropriate responses that do not induce autoimmunity, and [3] be safe to administer in humans. Previous preclinical immunogenicity and protection studies have demonstrated that the antigens are able to activate B-cells and elicit production of p*17 and K4S2-specific antibodies that are long lasting, have a high binding efficiency to the surface of *S. pyogenes* isolates, protect against several *S. pyogenes* strains and are not toxicologically adverse^4,5,24,28,29^. The current study has demonstrated that the vaccine antigens do not induce host tissue cross-reactive antibodies or autoimmune and inflammation pathology. Based on this body of evidence, we expect that a vaccine containing P*17 and K4S2 will be safe to administer in clinical trials.

With the advent of the recently characterised *S. pyogenes* controlled human infection challenge model, we can now assess safety and efficacy of streptococcal treatments in the clinical trial setting^30^ with healthy volunteers. The aim is to extend all the tools available to us to assess the utility of the vaccines in preventing streptococcal infection in the 3–8% of any population that are genetically susceptible and go on to develop chronic conditions like ARF, RHD and SC. The RAV model used in this study is a preclinical tool that is advancing our ability to achieve that aim.

## Methods

### Antigens

Lyophilised peptides p*17 and K4S2 were commercially sourced (China Peptides Co.) and conjugated to diphtheria toxoid (DT) (Chengdu Olymvax Biopharmaceuticals Inc.) using 6′-maleimido-caproyl *n*-hydroxy succinimide (MCS). Briefly, DT was first dialysed against 0.1 M phosphate buffer pH 7 using an Amicon ultracentrifuge tube (10 kDa). The DT concentration was then determined by Pierce™ Bicinchoninic acid protein assay kit (BCA) (ThermoFisher Scientific, Australia) and a working solution of 10 mg/mL was made. To activate the DT, a 10-molar excess of N-[ε-maleimidocaproyl] succinimide ester (MCS) in Dimethylformamide (DMF) was added to the dialysed DT. The solution was mixed slowly for 1 h at room temperature. The activated carrier was then dialysed against 0.1 M phosphate buffer pH 7 containing 0.1 M EDTA using a dialysis bag (10 kDa). After 2 h the buffer was renewed and then left mixing overnight, to remove excess MCS and DMF. Peptide was then conjugated to activated DT in a 1.2 M excess. Activated carrier was added directly to a known weight of lyophilised peptide and allowed to react at room temperature for 1 h with slow mixing. Peptide-conjugate was then dialysed overnight against 1X PBS^31^. Visual confirmation of conjugation was by SDS-PAGE (4–15%) analysis. Protein concentration determined by BCA. Recombinant M-protein of *S. pyogenes* (rM5) was prepared^32^. Briefly, the *S. pyogenes* M5 protein gene was cloned and expressed in *E. coli* BL21. His-tagged recombinant M5 proteins were purified by Ni-NTA resin (Qiagen, Australia) and visualised using SDS-PAGE. Protein was purified by buffer exchange using 1× PBS (pH 7.4) in a chromatography column and concentrated by ultrafiltration. Protein concentration was determined using the Bradford assay (Bio-Rad, Australia).

### Experimental animals

All the study protocols were approved by the Animal Ethics Committee of the University of New England (UNE) (AEC21-025). Female Lewis rats (LEW/SsN; Albino:a,h,c:RT^1^) age between 4–6 weeks were obtained from Centre for Animal Research and Teaching at UNE.

### Injection of rats

Prior to injection, Lewis rats (4–6 weeks old, *n* = 6) were anaesthetised by isoflurane inhalation (5% induction and 3% maintenance) in 0.5 L/min oxygen flow rate^13,33^. On day 0, rats received a 200 µL subcutaneous injection into the hock, of recombinant M5 (rM5) (500 µg; positive control), PBS (100 µL; negative control), P*17-DT (150 µg) or K4S2-DT (150 µg) mixed 1:1 with Complete Freund’s Adjuvant (CFA; Sigma, Australia). On days 1 and 3, rats were injected intraperitoneally with 0.3 µg of *Bordetella pertussis* toxin (Sigma, Australia) in 200 µL of PBS as an additional adjuvant^32–35^. All rats received 200 µL booster injections of antigen emulsified 1:1 with incomplete Freund’s adjuvant (IFA; Sigma, Australia) on days 7 and 14. On day 21, final booster was administered as follows; rM5 and PBS (in IFA) were injected subcutaneously into the flank region and P*17-DT and K4S2-DT were administered intranasally mixed in 200 µL of Tris.

### Behavioural assessments

Behavioural tests were performed to assess impairment in fine motor control (food manipulation), gait and balance (beam walking), obsessive-compulsive behaviour (grooming and marble burying) and anxiety-like behaviour prior to injections and cull. Behavioural tests were performed as briefly described below^36^. Assessments of all behavioural tests were blinded.

#### Food manipulation

Rats were deprived food for 24 h then provided with two pellets of rodent laboratory chow. The ability to manipulate the food for 10 min was blind-rated independently by two observers, using the Kolb and Holmes scale^37^.

#### Beam walking

Motor coordination and balance was measured as the ability of rats to traverse a narrow (2.5 cm) square wood beam mounted on a narrow support, elevated 30 cm above the ground. Time taken to traverse the narrow beam and the number of foot slips were recorded^38^.

#### Grooming

Individual rats were placed in a plexiglass observation box and misted with water to induce grooming. Duration of grooming bouts was assessed for 20 min immediately after misting^39^.

#### Marble burying

Individual rats were placed in a cage containing Corncob bedding and nine marbles arranged in two rows. The number of marbles buried or covered half or more by the bedding over a 15-min period was assessed.

#### Light/dark zones

A box containing light and dark zones was used to ascertain anxiety-like and exploratory behaviours. Rats were placed in the dark zone of the box and latency to move into the light compartment, time spent in the light compartment and number of entries into the light compartment time was measured for 5 min using ANYmaze software (Stoelting Co., Wood Dale, IL, USA).

### Electrocardiography (ECG)

ECG was performed under isoflurane inhalation anaesthesia in all rats prior to injection and a day before culling to assess conduction abnormalities in the heart. The electrical potentials were recorded for 1–2 min with LabChart 8 software provided by AD Instruments (Power Laboratories). Peak values of P and R points at three different segments of ECG from each rat was individually extracted and analysed^11^^,14,36^.

### Euthanasia of rats and collection of blood and tissue samples

Rats were culled day-35 post primary injection by intraperitoneal injection of sodium pentobarbital (260 mg/Kg). Blood was collected immediately following death via cardiac puncture using a needle fitted with a 5 mL heparinized syringe. Heart and brain tissue were collected following transcardial perfusion with PBS followed by 10% buffered formalin for 15 min. All tissue samples were fixed in 10% formalin overnight for histology.

### Histology and immunohistochemistry of cardiac tissues

Formalin-fixed heart tissue was processed, embedded in paraffin, sectioned and stained with Haematoxylin & Eosin (H&E)^14^. Slides were examined microscopically for infiltration of inflammatory cells as evidence of myocarditis or valvulitis. The extent of inflammation was expressed as a “carditis score” based on the number of inflammatory cells and focal lesions from each rat^14^. The scoring system assesses and scores valvular and myocardial sections (5 randomly selected areas per animal) from the different groups. For cytokine staining, formalin-fixed and paraffin-embedded sections (5 μm) were deparaffinized in xylene and rehydrated with graded ethanol. All the incubation steps were done at room temperature (RT) in a humidified chamber and the slides were washed thrice for 5 min with 10 mM Tris-buffered saline (TBS) at pH 7.4. Epitope retrieval was done with 10 mM Tris-EDTA (pH 9.0) for 15 min at 700 W in a laboratory microwave. Sections were incubated in 3% H_2_O_2_ in TBS for 10 min to inhibit endogenous peroxidase activity prior to being blocked with 10% normal sheep serum in TBS for 1 h. Blocking buffer was removed and slides incubated with rabbit anti-rat IL-17A at 1:100 (ab214588, Abcam, USA) overnight at 4 °C in a humidified chamber. Sections were washed and incubated with biotinylated donkey anti-rabbit IgG 1:500 (711-065-152, Jackson ImmunoResearch, USA) for 2 h. Slides were rinsed in TBS and sections were incubated with avidin–biotin complex (PK-6100, VECTASTAIN Elite ABC-Peroxidase Kit) for 1 h. Following final incubation sections were washed and developed using 3,3′-diaminobenzidine (DAB) substrate (Sigma, St Louis, MO, USA) and counterstained with Harris haematoxylin.

### Immunogenicity

Sera antibody titres to specific peptides was quantified using ELISA^5^. Briefly, antigens p*17, K4S2 (5 µg/mL) or rM5 (1 μg/mL) were coated onto Nunc Maxisorp plates (ThermoFisher Scientific, Australia). After overnight blocking with 5% skim milk in 0.05% PBS Tween-20, controls and test sera were applied (as serial dilution 1:2 starting at 1:100 to 1:3276800) diluted in 0.5% skim milk in 0.05% PBS Tween-20. After washing, goat anti-rat HRP (5204-2504, Bio-Rad, USA) secondary antibody was applied at 1:10,000 dilution. Plates were developed with o-Phenylenediamine dihydrochloride) tablets (OPD) (Sigma, Australia) and OD measured at 450 nm. IgG titres were defined as the highest dilution of serum for which OD was >3 standard deviations above the mean OD of PBS control at 1:100 dilution.

### Detection of tissue-reactive serum antibodies

Cross-reactivity of serum IgG, at fixed dilution of 1:400, against antigens p*17 and K4S2 were assessed by ELISA. Briefly, antigens p*17, K4S2 (5 µg/mL) or rM5 (1 μg/mL) were coated onto Nunc Maxisorp plates (ThermoFisher Scientific, Australia). After overnight blocking with 5% skim milk in 0.05% PBS Tween-20, test sera were applied at a fixed dilution of 1:400 diluted in 0.5% skim milk in 0.05% PBS Tween-20. After washing, goat anti-rat HRP (5204-2504, Bio-Rad, USA) secondary antibody was applied at 1:10,000 dilution. Plates were developed with o-Phenylenediamine dihydrochloride) tablets (OPD) (Sigma, Australia) and OD measured at 450 nm. Cross-reactivity of serum IgG against host proteins cardiac myosin, laminin, tropomyosin, dopamine receptors 1 and 2, lysoganglioside and tubulin and antigen rM5 were assessed by ELISA^14^. Briefly, antigens were coated onto Nunc Maxisorp plates (ThermoFisher Scientific, Australia) at the concentration 1 μg/mL of rM5 and 10 μg/mL of cardiac myosin, laminin, tropomyosin (Sigma, Australia), dopamine receptor 1 and 2 (Aviva Systems Biology, USA), lysoganglioside (Sigma, Australia) and tubulin (MP Biomedicals, USA). After blocking with 1% bovine serum albumin, individual rat sera were added at the concentration of 1:400. The secondary antibody goat anti-rat IgG-HRP (112-035-003, Jackson ImmunoResearch, USA) was added at 1:5000 dilution. Plates were developed with, 2-2′-azino-di(3-ethylbenzthiazoline)-6-sulphonate (ABTS) (Sigma, Australia) and OD measured at 415 nm.

### Detection of brain tissue reactive serum antibodies by western blot

Western blot was performed to assess the immune reactivity of rat sera with endogenous proteins from brain homogenate. Tissue from naive Lewis rats including the striatum and cerebellum from brain and kidney (non-neuronal control) were dissected. Tissues were homogenised with cold PBS with 1 mM EDTA in bead bug (Sigma, USA) for 1 min at 3000 × *g*. Following homogenisation RIPA buffer and protease inhibitor cocktail was added and centrifuged at 15,000 × *g* for 15 min at 4 °C. The supernatant was collected an aliquot of each sample was used to determine the protein concentration by Bradford Assay (Bio-Rad, Australia). Twenty micrograms of each tissue lysates from striatum, cerebellum and kidney were separated using 10% non-reducing sodium dodecyl sulfate-polyacrylamide gel electrophoresis (SDS-PAGE), and the fractionated proteins transferred to a Polyvinylidene fluoride (PVDF) membrane using Trans-Blot®Turbo™ Transfer System (Bio-Rad, USA) using transfer buffer. Following transfer, the PVDF membrane was blocked with 1% BSA in TBS for 1 h. The membrane was washed in Tris-buffered saline containing 0.1% Tween® 20 (TBST) for 2 × 5 min and incubated overnight at 4 °C with 5 mL pooled serum (dilution ratio of 1:400) using an end over end mixer. The membranes were washed 4 times with 30 mL of TBST at room temperature with gentle agitation and incubated with a 1:10,000 horseradish peroxidase (HRP) conjugated goat anti-rat IgG secondary antibody (112-035-143, Jackson ImmunoResearch, USA) at room temperature for 1 h. Following incubation membrane was washed 8 times with 30 mL of TBST and developed with Clarity Western ECL substrate (Bio-Rad, Australia). Excess substrate was blotted from the membrane and a chemiluminescence and a light image were acquired using ChemiDoc™ Imaging System (Bio-Rad, Australia). Membrane with tissues lysates were stripped using stripping buffer and re-incubated with 1:1000 GAPDH Rabbit monoclonal antibody (2118, Cell Signaling Technologies, USA) to determine the expression of housekeeping proteins and developed with substrate. Uncropped blots are provided in Supplementary Information (Supplementary Figure 2).

### Detection of IL-17A in serum

Detection of cytokine IL-17A in rat sera was conducted with the ELISA MAX™ Deluxe Set Rat IL-17A kit (BioLegend). Briefly, sera from all rats in a group was pooled and 100 µL of neat, pooled sera, in duplicate, was analysed using the IL-17A kit. Absorbance measurements were taken at 450 nm and 570 nm on an Infinite 200 Pro spectrometer (Tecan) using Tecan i-control software. Absorbance at 570 nm was subtracted from the absorbance 450 nm as per manufacturer’s instructions.

### Statistical analysis

Descriptive statistics of results were made using GraphPad Prism version 8.0. Antibody titre data are presented as geometric mean. One-way ANOVA with Dunnett post hoc method for multiple comparisons was used for pairwise comparisons. Results were taken as significant with *P-*values <0.05 or <0.01. Two-way ANOVA was used to assess behavioural and ECG differences between rats. All data were reported as means ± SEM and *P*-values <0.05 were considered significant.

### Reporting summary

Further information on research design is available in the Nature Research Reporting Summary linked to this article.

## Supplementary information

Supplementary Information

REPORTING SUMMARY
