# Supplementary material for: Streptococcus pyogenes vaccine candidates do not induce autoimmune responses in a rheumatic heart disease model
Source: NPJ Vaccines. 2023 Feb 4;8:9. doi: 10.1038/s41541-023-00604-2 (PMC9899064; doi:10.1038/s41541-023-00604-2)
Supplement: Supplementary file 1 — Supplementary Information [file 41541_2023_604_MOESM1_ESM.pdf]

| Score | 0 | 1 | 2 | 3 | 4 |
| --- | --- | --- | --- | --- | --- |
| Myocardium<br><br>(X200) | Diffuse, individual cells throughout tissue<br>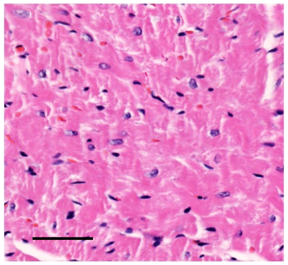 | 1-2 small foci<br>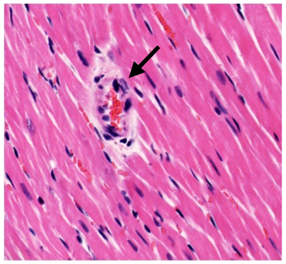 | >2 small foci<br>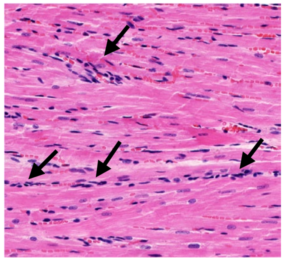 | Large focal lesion<br>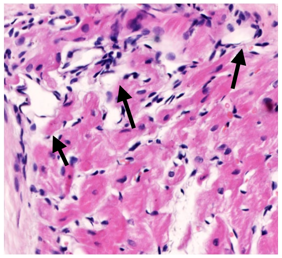 | Aschoff-type lesion<br>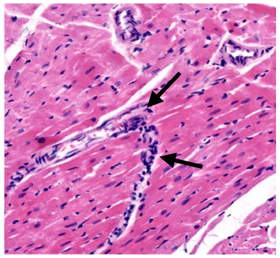 |
| Mitral valve<br><br>(X200) | No inflammatory cells associated with valves<br>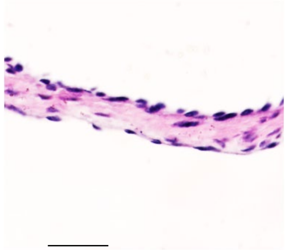 | <5 isolated cells in/on valves<br>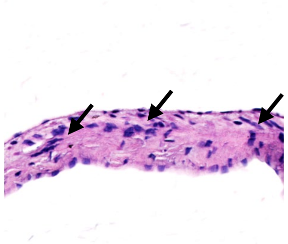 | >5 cells on valve surface only<br>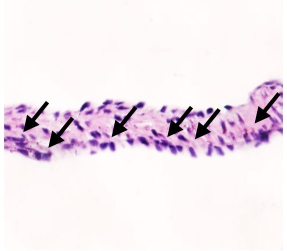 | Focal lesion in valve<br>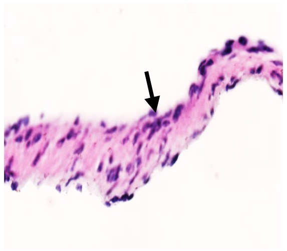 | >1 lesion<br>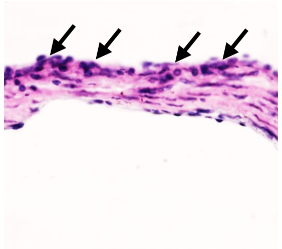 |

**Supplementary Table 1.** Carditis score. Formalin fixed paraffin embedded sections were stained with haematoxylin and eosin (H&E) using standard protocols. Stained tissue were examined for evidence of valvulitis and myocarditis. Observation under microscope of inflammatory cell infiltration into tissue was performed double blinded using 5 different sections of the myocardium and the mitral valve from each rat. Individual scores (0-4) for both myocardial and valvular tissue were summed to derive at the "carditis" score.  Scale bar represents 50μm.

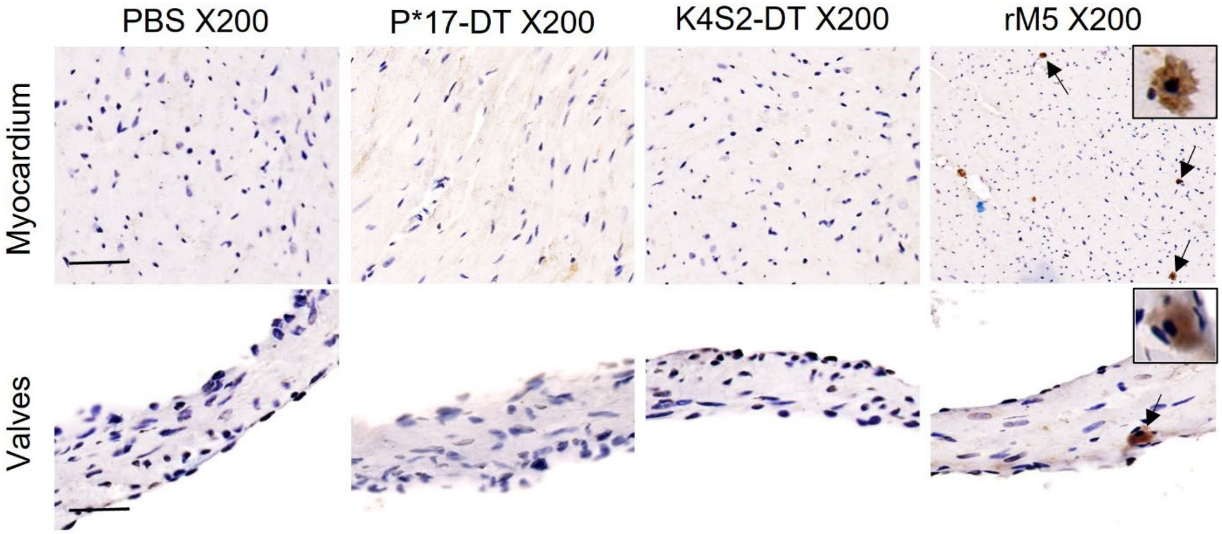


**Supplementary Figure 1.** Tissue specific localisation of IL-17A. Presence of IL-17A in the myocardium and valvular tissues of rats was investigated by immunohistochemical staining. No evidence of cellular infiltration of IL-17A was observed in the PBS control, P*17-DT and K4S2-DT treated rats. In contrast, IL-17A was detected in rM5 treated rats (arrows). Representative images from two rats in each group given. Images magnified at x200, snippets magnified at x400 and scale bar represents 50 μm.

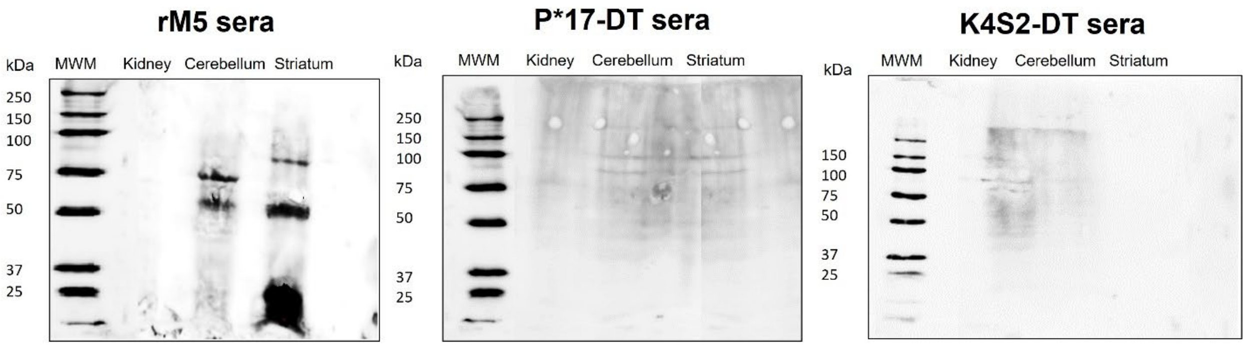


**Supplementary Figure 2.** Serum IgG cross-reactivity to host brain tissue. Serum IgG cross reactivity to endogenous proteins in lysates of cerebellum, striatum and kidney

(none-neuronal control) from naïve rats. Uncropped blots shown. MWM; molecular weight marker.
